# Operating to Cure Criminal Impulses

**Published:** 1914-08

**Authors:** 


OPERATING TO CURE CRIMINAL IMPULSES.—It has long been recognized by physicians that much of the crime of the day is the result of diseased brains, and that many criminals should be sent to sanitariums for treatment instead of to prisons. Operations that result in the removal of pressure on the brain are frequently performed to cure insanity of certain forms, and if certain criminal tendencies result from insanity it stands to reason that criminal inclinations may be cured by operations in certain cases. The following story from Canyon City, Colorado, published recently in the daily press, may lead to a closer study of the extent to which crime is related to disease:

"A splinter of bone lifted from his brain, and Jeff Lee, prisoner in the State penitentiary here, has been transformed from a criminal maniac to an honest man. Still in his early 20's, the surgeon's knife has wrought a miracle that will send the young man into the world a free man in every sense of the word—free from the penalty of the law, and free, also, from the malign influence that made him a thief and all-round petty criminal.

"A fall from a horse, when he was only 15 years old, made of him a criminal, a vagrant, and finally a maniac. The removal of the splinter of bone has relieved the pressure on the brain that short-circuited the nerve currents and perverted the lad's impulses and acts.

"Jeff Lee is the son of a wealthy Texas planter. He was a healthy, normal youth, with a good record in his studies, and with many friends. When he was 15 several of his boy friends dared him to ride an unruly horse. He accepted the challenge and apparently conquered the beast after a sharp struggle. Then he invited one of his girl friends to accompany him for a ride. They rode off together and, while they laughed and chatted, the horse, seeing an opportunity, suddenly reared and threw the youth. He was dashed violently to the ground, sustaining a fractured skull. He was taken home, and recovered, so far as his physical health was concerned.

"But from that time he grew morose and sullen, in striking contrast to his former unvarying good nature. Then he began to seek the company of the roughs of the neighborhood, and to practice evil ways. Finally he was arrested and convicted on a charge of horse stealing. He went to the Texas penitentiary, and there, with the hardened criminals as his willing tutors, he soon became versed in crime. When he was released from the Texas prison he came to Colorado and soon became intimate with a criminal element. In a few months he was arrested on a charge of burglary. Lee was sentenced to from two to five years in the State prison. His second commitment seemed to prey on his mind. He showed signs of incipient insanity almost from the day of his entry, and within a few months was sent to the ward for the insane, having become apparently a total maniac, with spells of violence.

"The surgeon of the penitentiary became interested in the case, and believing an operation would restore his reason, and possibly eliminate his criminal tendencies, an effort was made to locate his relatives, so that their permission might be obtained. The prisoner, however, had concealed his true name, and for a time efforts were fruitless. Then one day a lucid interval came. Lee, recognizing his plight, gave the names of his parents, and begged that the operation be performed. "I would rather die this minute than to go on living as a crook," he said. The parents gave their consent. Four months ago the operation was performed. When Lee recovered from the anesthetic he was sane. Today he stands upright, with the straightforward gaze of the honest man. In a few months, when his minimum sentence expires, he will be released."

